# Methane Exchange in a Coastal Fen in the First Year after Flooding - A Systems Shift

**DOI:** 10.1371/journal.pone.0140657

**Published:** 2015-10-13

**Authors:** Juliane Hahn, Stefan Köhler, Stephan Glatzel, Gerald Jurasinski

**Affiliations:** 1 Landscape Ecology, Faculty of Agricultural and Environmental Sciences, University of Rostock, Rostock, Germany; 2 Department of Geography and Regional Research, University of Vienna, Vienna, Austria; St. Francis Xavier University, CANADA

## Abstract

**Background:**

Peatland restoration can have several objectives, for example re-establishing the natural habitat, supporting unique biodiversity attributes or re-initiating key biogeochemical processes, which can ultimately lead to a reduction in greenhouse gas (GHG) emissions. Every restoration measure, however, is itself a disturbance to the ecosystem.

**Methods:**

Here, we examine an ecosystem shift in a coastal fen at the southern Baltic Sea which was rewetted by flooding. The analyses are based on one year of bi-weekly closed chamber measurements of methane fluxes gathered at spots located in different vegetation stands. During measurement campaigns, we recorded data on water levels, peat temperatures, and chemical properties of peat water. In addition we analyzed the first 20 cm of peat before and after flooding for dry bulk density (DBD), content of organic matter and total amounts of carbon (C), nitrogen (N), sulfur (S), and other nutrients.

**Results:**

Rewetting turned the site from a summer dry fen into a shallow lake with water levels up to 0.60 m. We observed a substantial die-back of vegetation, especially in stands of sedges (*Carex acutiformis* Ehrh). Concentrations of total organic carbon and nitrogen in the peat water, as well as dry bulk density and concentrations of C, N and S in the peat increased. In the first year after rewetting, the average annual exchange of methane amounted to 0.26 ± 0.06 kg m^-2^. This is equivalent to a 190-times increase in methane compared to pre-flooding conditions. Highest methane fluxes occurred in sedge stands which suffered from the heaviest die-back. None of the recorded environmental variables showed consistent relationships with the amounts of methane exchanged.

**Conclusions:**

Our results suggest that rewetting projects should be monitored not only with regard to vegetation development but also with respect to biogeochemical conditions. Further, high methane emissions that likely occur directly after rewetting by flooding should be considered when forecasting the overall effect of rewetting on GHG exchange.

## Introduction

The world’s peatlands play an important role in the global climate system with regard to the exchange of greenhouse gases (GHG) [1]. They constitute the largest and most concentrated reservoir of carbon (C) of all terrestrial ecosystems, storing worldwide an estimated 550 Gt of C in their peat [2]. They further provide a habitat for many characteristic and some highly adapted plant and animal species [3].

Draining and land use of drained peatlands not only change their plant and microbe community composition [1] but can also transform these C stores into C sources e.g., [4, 5]. Thus, drainage for agricultural use has a lasting effect on the C balance of wetlands [6, 7]. In addition drainage irreversibly alters chemical and physical characteristics of the peat [8, 9].

Until the 1980s the use and drainage of peatlands was the primary objective of peatland management in Europe and Northern America but the restoration of altered peatlands has gained importance over the last three decades [10]. Initially, restoration of peatlands primarily aimed at biodiversity protection. However, the reduction of GHG emissions has grown as an objective of peatland restoration [11]. Today peatland rewetting and/or restoration seem to be common land-use management practices [12] in Europe and Northern America. This is also acknowledged in the recent Wetland Supplement of the IPCC report [5].

A systematic review of the exchange of GHG in restored peatlands is not available at the moment [10] although some information can be gained from the IPCC Wetland Supplement and the chapter on Rewetted Organic Soils [5]. The storage of C is determined by the balance between primary production (photosynthesis) and decomposition [13]. Raising the water level in drained peatlands has the potential to decrease aerobic decomposition, and under certain conditions is expected to reduce net GHG emissions [14, 15, 16]. Therefore, the re-establishment of hydrological self-regulation and peat accumulation are key aspects of efforts to restore the C sink function of peatland ecosystems [17, 18]. However, very few studies really compare full GHG balances before and after flooding under field conditions [19]. The available data suggest, though, that CH_4_ emissions may increase after rewetting whilst CO_2_ and N_2_O emissions are effectively reduced [20] but this is deduced from measurements in drained, pristine and very few rewetted peatlands, e.g., [10, 21]. Data on the effects of rewetting of peatlands on ecosystem functioning including GHG exchange, vegetation succession as well as peat and water chemistry are even more sparse—but see [12] for some references addressing nitrogen compounds and dissolved organic C.

GHG emissions in peatlands are modulated by complex relationships between a range of interconnected biological, chemical and physical factors [22]. Even within one peatland the relationships between water level, decomposition and sequestration are not necessarily straightforward [23]. Furthermore, there is disagreement in the interpretation of the variables, reactions and the impact of environmental conditions on C storage and release. Thus, a better understanding of the critical processes regulating GHG dynamics in peatlands in general and particularly under flooded conditions is needed [24].

In boreal peatlands, restoration after peat mining in cut-over bogs is quite common, e.g., [25, 26, 27]. Restoration of fens has been less widespread [10] but during the last decades 10 000 ha of drained, lowland fens have been rewetted in Northeast Germany [28] and 2 500 ha in Northwest Germany (NLWKN (2007), in [10]). When low lying fens are rewetted, they are often flooded since they subsided under drainage because of compaction, shrinking and decomposition with rates of up to 2cm a^-1^ in temperate and boreal peatlands [29, 17] resulting in ground surface levels below sea level or local ground water table. When rewetting is then achieved by stopping pumping or by blocking the main drainage ditches at the outflow, flooding is inevitable. With regard to biogeochemical cycles flooding leads to increased anoxia within the peat profile coupled with increased substrate supply in terms of previously mineralized organic matter and plants dying from inundation. This is likely to fuel anaerobic degradation and thus CH_4_ production; especially at high temperatures [30].

When land managers plan rewetting projects they want to know beforehand whether their measures will be effective. Plant species and vegetation composition may help in predicting future emissions after rewetting. Dias et al. [31] have introduced plant species composition as a proxy to predict CH_4_ emissions in peatland ecosystems after land-use changes. Similarly, Couwenberg et al. [32] have developed the methodology of Greenhouse gas Emission Site Types (GESTs) to assess emissions and emission reductions from peatland rewetting projects using vegetation as a proxy [32]. Couwenberg and Fritz [33] stated that vegetation is a good proxy for mean water levels and could provide—with extra attention to species with aerenchyma tissue (“shunt species”)—a robust proxy for accurate and spatially explicit estimates of CH_4_ effluxes over large areas. This only holds true, however, when the vegetation already has adapted to the rewetted conditions. Another major problem in forecasting GHG emissions after rewetting, is the considerable variation in starting conditions (i.e. genesis and succession of the site followed by the history of anthropogenic alterations) [10] and the lack of knowledge about changes regarding water quality and peat chemistry and their impact on changes in GHG exchange rates.

Here, we study the initial rewetting phase in a brackish fen at the coast of the southern Baltic Sea. During the first year after flooding, we investigated vegetation development, peat and water properties relevant to ecosystem functioning and the emission patterns of CH_4_. We hypothesized that flooding will cause (1) an increase in CH_4_ emissions because water level is the strongest driver of CH_4_ emissions in temperate peatlands, (2) a die-back in the vegetation because the prevailing plants are adapted to non-flooding conditions and (3) a shift in peat and water properties because the inflow of fresh water from the catchment will very likely alter the biogeochemical status and the associated processes.

## Materials and Methods

### Study site

**Fig 1:**
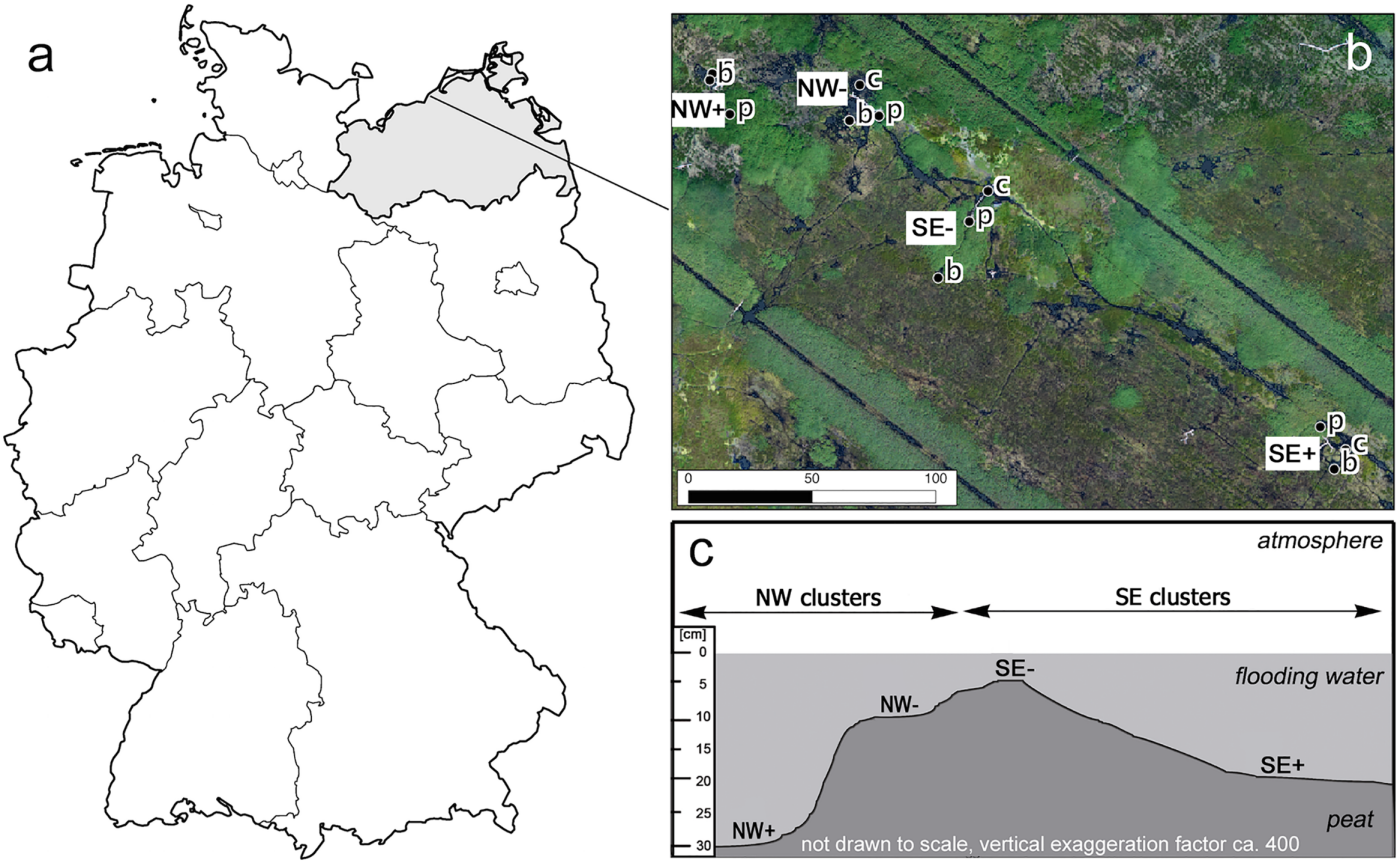
(a) Location of the study site in northeast Germany. (b) Spatial distribution of the twelve measurement spots at the four clusters (aerial photograph 2009, NW = northwest, SE = southeast, + = more inundated, − = less inundated, b = rush stands, c = sedge stands p = reed stands). (c) micro-relief and inundation intensities in November 2009 (NW = northwest, SE = southeast). Own creation. Map in a) is reprinted from Bundesamt für Kartografie und Geodäsie under a CC BY license, with permission from the German Ordinance to Determine the Conditions for Use for the Provision of Spatial Data of the Federation (GeoNutzV), original copyright 2014.

The study site “Rodewiese” is part of a paludification fen that is located at the southern shore of the Baltic Sea close to the city of Rostock, Northeast Germany (lat 54°21’N, long 12°18’E) in the nature reserve “Heiligensee und Hütelmoor” (Fig 1a and 1b) [34]. Access to the site was granted by the Forest authority of the city of Rostock (Stadforstamt). The site covers an area of about 0.63km². The climate is temperate with an average annual temperature of 9.1°C and an average annual precipitation of 645 mm [35] (reference climate period: 1981–2010). The nature reserve is separated from the Baltic Sea by a dune dike. In the past, it was episodically flooded with brackish waters. The last major intrusion took place in 1995 [36].

Formerly, the site had been drained by a network of ditches. From 1975 drainage was intensified and soil was ploughed up and mixed with sand at 70% of the area in order to use it for forage crops (land amelioration) [37, 38]. Starting in 1990, several restoration measures were implemented. Drainage was stopped in 1992 what led to slight rewetting of the “Rodewiese”. To prevent the water level to drop considerably below ground surface in summer, a groundsill was installed at the outflow of the catchment in late 2009 [39]. Already some years before that, in 2005, the maintenance of the dune dike was abandoned.

The soil at the study site is a sapric histosol consisting of 1–3 m thick layers of alternating sandy sediments and horizons of reed-sedge peat. Due to intense drainage between the 1970s and 1990s the peat layers are highly degraded (up to H10 at the von Post humification scale). The vegetation of the study site consists of a dynamic mosaic of reed, rush and sedge stands featuring species that are adapted to temporal variation in brackishness. The four most important species that form dominance stands are Common reed (*Phragmites australis* L.), Lesser pond sedge (*Carex acutiformis* Ehrh.), Sea club-rush (*Bolboschoenus maritimus* (L.) Palla.) and Softstem bulrush (*Schoenoplectus tabernaemontani* C.G. Gmel.). The latter two often occur together with shared dominance.

In 2009, before flooding, the site was a weak source of CH_4_ with average annual emissions of 13.94±5.8 kg ha^-1^ a^-1^ CH_4_ [34] and a sink for CO_2_ with average uptake of 12.2 ± 0.05 t ha^-1^ CO_2_ during the growing season (May-October) 2009 [39]. Emissions of CH_4_ varied between different vegetation types with significantly higher fluxes (31.8±5.7 kg ha^-1^ a^-1^) from rush stands with *Bolboschoenus maritimus* compared to sedge stands (4.3±1.2 kg ha^-1^ a^-1^). None of the other tested variables (root density, pH, ash content, mean annual water level, mean annual conductivity) had a significant influence on the variation in CH_4_ emissions [34].

### Field setup

**Fig 2:**
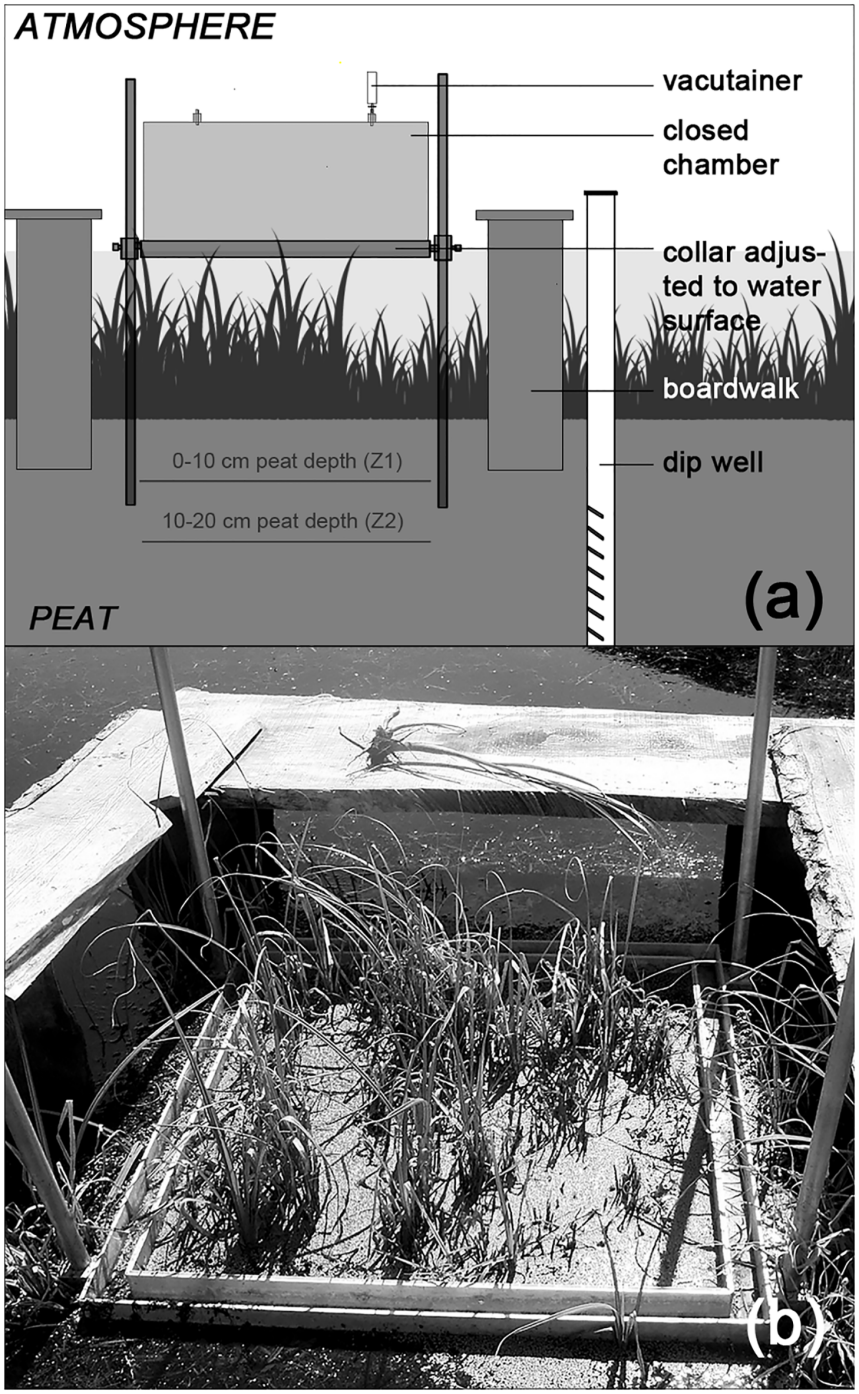
Closed chamber set-up to measure the CH_4_ exchange at inundated conditions. a) Scheme of a measurement spot with the chamber placed at the collar and an attached vacutainer for gas sampling. b) Photograph of a sedge spot from the study site in July 2010.

In October 2009, 12 measurement locations (spots) were set up. Each three of these, covering different dominant vegetation (Common reed (“reed”), Lesser pond sedge (“sedge”) and Sea-club rush / Softstem bulrush (“rush”)) were arranged in clusters at two inundation levels (one less (-) and one more (+) inundated). This was replicated in two independent areas (locations) of the study site (NW and SE). We permanently installed aluminum collars at these spots four weeks before measurements started. The collars were held in place by aluminum rods allowing for height adjustment of the collars to the water table and were each surrounded by a wooden boardwalk to minimize disturbances during sampling (Fig 2).

For measuring water level and for gathering peat water samples we installed one to two dip wells per cluster (2.20 m depth, 35 mm internal diameter, PVC slotted screen, custom made) in November 2009 (Table 1). Percent cover of vascular plants was assessed in August 2010 within each measurement spot following [40]. Regular field sampling campaigns were run biweekly from November 2009 to November 2010. Thus, the first measurement campaigns were carried out before flooding, allowing us to derive pre-flooding data on peat and water properties.

**Table 1:** Average peat water properties at the NW and SE clusters in the first year after flooding.

location	WL (m)	EC (mS cm^-1^)	Cl^-^(g l^-1^)	SO_4_^2-^ (g l^-1^)	TOC (g l^-1^)	TNb (mg l^-1^)	n
**NW**	0.39 (0.1)***	5.37 (2.3)	0.79 (0.7)	0.32 (0.3)	0.10 (0.1)	6.20 (4.9)	90
**SE**	0.32 (0.1)	6.42 (2.8)*	1.06 (1.2)	0.95 (1.7)***	0.12 (0.1)	7.70 (9.6)	109, WL = 120

Values are arithmetic means and standard deviations are given in brackets. Differences between NW and SE clusters were tested with one-tailed Wilcox-rank-sum-tests and significance is indicated by:

* p<0.05 and,

*** p<0.001.

WL = water level above ground, EC = electric conductivity, Cl^-^ = concentration of Cl^-^ anions in the peat water, SO_4_^2-^ = concentration of SO_4_^2-^ anions in the peat water, TOC = total organic carbon and TN total bound nitrogen in the peat water; NW = northwest; SE = southeast.

### Peat water properties

During field sampling campaigns we recorded water table levels, water temperatures, electric conductivities, and pH-values in the dip wells at a depth of 10 cm below water surface (Universal Pocket Meter (Multi340i), Wissenschaftlich-Technische Werkstätten GmbH (WTW), Weilheim, Germany). Water samples were taken monthly with syringes from the dip wells from 10 cm below the water surface to be analyzed for anions with importance in brackish systems (Cl^-^ and SO_4_^2-^), for total organic carbon (TOC), and for total bound nitrogen (TNb). TOC and TNb were determined to assess the nutrient content of the water phase.

The water samples were transported to the lab cooled and stored at -20°C until analysis with ion chromatography. To prepare the samples for anion analysis a pinch of sample-preparation-resin D7 (Frank Gutjahr Chromatographie, Balingen, Germany) was mixed with 60 ml of sample to remove humic acids. The mixture was filtered over a folded filter (Art. CA08.1, Carl Roth, Karlsruhe, Germany). Filtrates were diluted with high-purity water according to their EC. Anion concentrations of the diluted filtrates were determined by ion chromatography (M IC, Metrohm GmbH & Co. KG, Filderstadt, Germany) using an anion exchange column with chemical suppression (MetrosepA Supp5-150, Metrohm Germany) with a carbonate eluent (2 mM NaHCO_3_ and 1.3 mM Na_2_CO_3_). Anion concentrations in the standards were 0.5 mg l^-1^, 2.0 mg l^-1^, 5.0 mg l^-1^, 10.0 mg l^-1^, 20.0 mg l^-1^ and 30.0 mg l^-1^ of Cl^-^ and SO_4_^2-^, respectively. An aliquot of the water samples was transferred to glass vials and analyzed for TOC and TNb (DIMATOC^®^2000, Dimatec Analysentechnik GmbH, Essen, Germany; smallest standard 0.1ml l^-1^ carbon and nitrogen). Raw data on peat water properties are given in S2 Dataset.

### Peat properties

Peat temperatures at 10 cm below ground surface were recorded with data loggers (Hobo Temperature/Light Pendant UA-002-64, Onset Computer Corporation, Inc., Pocasset, Massachussetts, USA) half hourly from August 2010 until January 2011 at each cluster. Peat temperatures for the whole investigation period were derived based on a linear regression model of the available measurements against the water temperature data (p<0.001, R^2^ = 0.96, n = 77). This model was used to predict peat temperatures for the times not covered by the direct peat temperature measurements via the water temperature data that covers the whole investigation period.

Intact peat cores (7 cm diameter) were collected from the top 20 cm of peat adjacent to the measurement spots (maximum 1 m apart) for detailed peat characterization in autumn 2009 (pre-flooding) and late summer 2010 (post-flooding). The upper (0–10 cm, D1) and lower (10–20 cm, D2) parts of the peat cores were analyzed separately to determine differences between the zone dominated by living roots (D1) and the more humified peat (D2). The samples were analyzed for dry bulk density, content of organic matter and total amounts of carbon (C), nitrogen (N), sulfur (S), as well as of phosphorus (P), magnesium (Mg), potassium (K), calcium (Ca), sodium (Na) and iron (Fe). Unfortunately, pre-flooding samples from cluster NW+ were lost because equipment broke during sampling and we were not able to repair it before the site was flooded after the ground sill was installed in late 2009.

Content of organic matter was estimated by loss-on-ignition. To do so, 200 g of fresh peat were dried at 105°C for 24 h and approx. 5 g of dried peat were incinerated in a muffle furnace at 500°C for 5 h (adapted from [41]). Dry bulk densities and element contents were determined on peat cores which were dried at 40°C for 48 h. One half of each core was subsequently dried at 105°C for 24 h to determine the bulk density by dividing the resulting dry mass by the volume of the half-core [42]. The second half of the 40°C dried cores was used to determine elements. The dried peat was powdered with a centrifugal ball mill (Typ 1, Retsch GmbH & Co. KG, Haan, Germany). The CNS-composition of the samples was determined by an Elementar analyser (VARIO EL, Elementar Analysensysteme GmbH, Hanau, Germany) according to Blume et al. [43]. To determine total amounts of P, Mg, K, Ca, Na and Fe ca. 30 g of dry peat were incinerated in a muffle furnace (550°C for 5 h) to remove organic material that could cause foam formation in the following extraction steps. 0.5 g of incinerated peat were then digested by 10 ml 65% HNO_3_ (p.a. grade) followed by a microwave-pressure-extraction (200°C for 15 min; Mars Xpress, CEM, Kamp-Lintfort, Germany). Ash-particles were filtered off and element-concentrations were determined by inductively coupled plasma optical emission spectrometry (ICP-OES) (Jobin Ivon JY238 ULTRACE, Horiba Jobin Ivon GmbH, Bensheim, Germany) at wavelengths 214.914 nm (P), 202.569 nm (Mg), 766.490 nm (K), 396.847 nm (Ca), 589.592 nm (Na) and 238.204 nm (Fe). Raw data on peat properties are given in S3 Dataset.

### Methane measurements

During field sampling campaigns we conducted chamber measurements as described in Koebsch et al. [34]. Atmospheric CH_4_-exchange (i.e. above-ground exchange) was determined based on opaque closed chamber measurements [44]. We used rectangular PVC-chambers with base areas of 0.5625 m^2^ and heights of 0.5 m (volume = 280 l). Two sampling outlets—made from flexible tubes held in place by cable glands and closed by stop cocks—were located at the top of the chamber. During measurement the chambers were placed in a 5 cm wide and equally deep channel of the preinstalled aluminum collars (0.75 * 0.75 m^2^). The channel was filled with water to provide an airtight seal during gas measurements (Fig 2a). We used chamber extensions with a height of 50 cm to allow for chamber measurements on tall emergent macrophytes.

During chamber measurements the three spots per cluster were measured in parallel. Gas samples were taken by evacuated glass-flasks (vacutainer, 46 ml) 0, 10, 20 and 30 min after chamber closure. Prior to each gas sampling we mixed the chamber volume by pumping with a 60 ml syringe for 1 min. Internal chamber temperature was recorded during chamber closure. The gas samples were analyzed within a week after field sampling with a gas chromatograph (PerkinElmer Auto System, Massachusetts, USA) equipped with a Porapak packed analytical column (Loftfields Analytische Lösungen, Germany). CH_4_ was detected by a flame ionization detector (FID) at 200°C with an average accuracy (deviation from the reference concentration) of 2% (149 ppb). Chamber headspace concentrations of CH_4_ are given in S1 Dataset.

### Methane flux calculation

CH_4_ fluxes were estimated from concentration change over time in the chamber headspace during chamber placement using package “flux” [45]) for R! [46]. The function *flux* of the package allows for automatic outlier detection and finds the best linear fit in the concentration data from closed chamber measurements by applying iterative linear regressions to the data points (see [34] for details). Fluxes that did not meet the set quality criteria (R^2^ > 0.8, NRMSE < 0.2) were excluded from further analyses. Many of them may have been ebullitions, however, since there is no straightforward method to distinguish between ebullitions and measurement errors we chose to omit fluxes based on our quality criteria alone. In addition, the range of the concentration measurements during chamber placement was compared to the daily repeatability range of GC-measurements. In case the range of the concentration measurements was smaller than the daily repeatability range, the respective flux was set to zero. Negative flux values indicate biospheric uptake of CH_4_ (sequestration of C), and positive flux values indicate CH_4_ emissions to the atmosphere. Annual emission estimates for each measurement spot were derived using a Monte Carlo permutation procedure provided by function *auc*.*mc* of the R package “flux” [45], for details see [47].

### Data analysis

All data analyses were performed using statistical software R! version 2.14.2 [46]. We interpolated CH_4_ fluxes and continuous environmental variables by generalized additive (GAM) models using function *gam* of base R. Plot wise GAMs were fitted to get fortnightly data sets of CH_4_ fluxes and the environmental variables. Fitting GAMs assumes linear developments between adjacent measurement dates in the respective variable. Especially for CH_4_ fluxes this assumption may be not fully justified because they can be highly variable in time [48]. Nevertheless, we obtained the possibility to display trends in the relationship between CH_4_ fluxes and environmental variables to illustrate their variability across space and time. Site wise GAMs were fitted for environmental variables to display trends during the measurement period.

Linear regression was applied to examine the development of CH_4_ fluxes and environmental variables (water level, TOC, TNb) during the measurement period. Furthermore, we used linear regression to investigate whether environmental variables are related (electric conductivity ~ anion content), whether they control CH_4_ fluxes (CH_4_ ~ temp, CH_4_ ~ TOC, TNb) and whether vegetation cover was driving atmospheric CH_4_ exchange over the study period (atmospheric CH_4_ exchange ~ percent cover of floating and rooted vegetation).

We used non-parametric statistics to account for (mainly) non-normal distribution of the data when comparing environmental variables and peat properties between locations, inundation levels, and clusters. For location and inundation level we used one tailed, paired, two-sample Wilcoxon signed rank tests with continuity correction, for clusters we used pairwise Wilcoxon rank sum test with Holm correction for p-values [49]. Both tests are not sensitive to normal distribution. In case the data were normally distributed, the Welch test was used instead. Significance level was set to p<0.05.

To examine changes of the peat water or peat properties before and after flooding as well as to jointly analyze relationships between annual CH_4_ exchange and site characterizing parameters we applied non-metric multidimensional scaling (NMDS) separately for water and peat. We used function “metaMDS” (package “vegan” version 2.0–5, [50] for R!) on transformed data (standardized into the range 0–1) with Euclidean distances and maximum 100 random starts in search for a stable solution. For peat water data we used water level, concentrations of TOC, TNb, chloride, and sulfate. For peat properties we included dry bulk density, dry mass, and concentrations of C, N, S, P, K, Mg, Ca, Na, Fe. For the analysis of the relationships between annual CH4 exchange and site characterizing parameters we used the averages of environmental variables from the measurement period, peat properties from late summer 2010 and data from the vegetation cover estimation. We excluded variables with a significance level below 0.05 by backward selection until the ordination results were constant. From this process, 13 parameters were retained, namely: electric conductivity and concentrations of TOC, TNb and SO_4_^2-^ in the peat water at 10 cm depth as well as dry bulk density, percent dry weight, content of organic matter and total concentrations of C, N, S, K, Mg and Na in the uppermost 20cm of peat. We checked the significance of the parameters that remained in the NMDS with a permutation test (function “envfit”, 1000 permutations; package “vegan” version 2.0–5, [50] for R!) and assumed that those with p<0.001 significantly characterized the measurement spots. Afterwards we used this permutation test to check for relationships of these site characteristics with the amount of CH_4_ exchanged during the first year after flooding. To visualize these relationships we scaled the symbols according to the amount of annual CH_4_ exchange.

## Results

### Site characteristics

#### Vegetation development

During the first year after flooding, we observed a substantial die-back of vegetation—especially in the sedge stands. This was reflected in the post-flooding plant species percent cover estimates in August 2010 compared to the pre-flooding estimates from August 2009: Percent cover of rooted vegetation ranged between 1 and 100%. Three of four spots with Lesser pond sedge showed the least cover of rooted vegetation compared to the other spots in the cluster. The actual sedge cover ranged from 0 to 20%. Furthermore, at ten out of twelve spots floating plants such as Duckweed (*Lemna* sp. L.) and Water starwort (*Callitriche* sp. L.) occurred during the study period. Percent cover of floating species significantly increased with decreasing cover of rooted vegetation (linear regression, n = 12, R² = 0.4445, p = 0.0179).

#### Peat water properties

**Fig 3:**
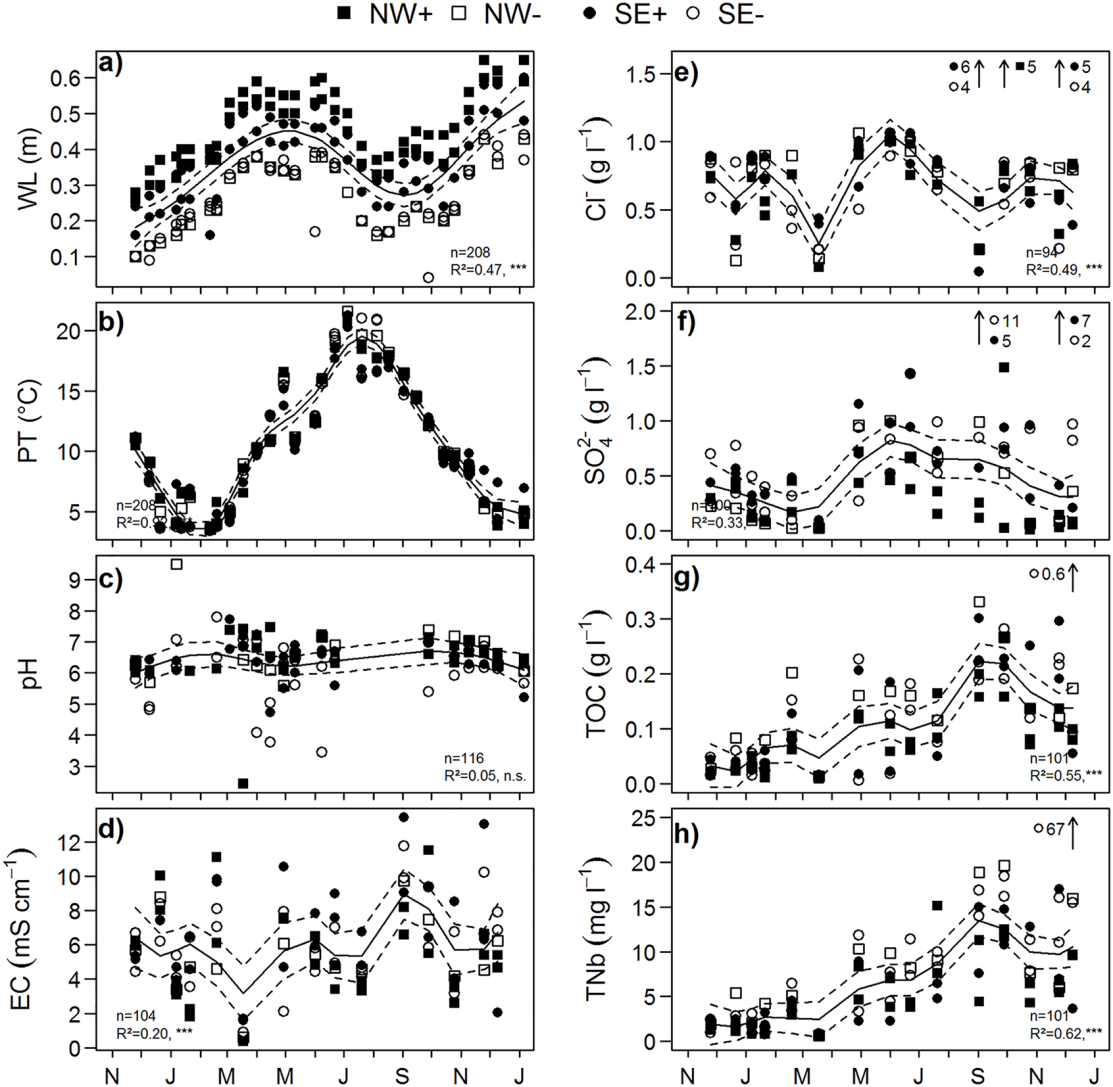
Environmental variables at the more (NW+, SE+) and less (NW-, SE-) inundated clusters after flooding. From November 2009 until January 2011, we recorded a) water level (WL), b) peat temperature (PT), c) pH-value, d) electric conductivity (EC), e) concentration of chloride (Cl^-^), f) sulfate (SO_4_^2-^), g) total organic carbon (TOC), and h) total bound nitrogen (TNb) in the peat water at 10 cm peat depth. Generalized additive models were fitted to the data to display the overall trends during the measurement period (solid line: modelled value; dashed lines: 95% confidence interval); the goodness of model fit is given by R^2^ and p-value. Extreme values (≥1.5 x interquartile range) were not included into modelling; they are marked by arrows and values are given next to the symbol of the particular cluster.

Due to the flooding of the area, the water levels increased significantly (linear regression, n = 208, R^2^ = 0.1128, p<0.001) from 17±8 cm (November before flooding) to 52±9 cm above ground surface (November after flooding). On average the increase amounted to 34 ± 1 cm and was most pronounced (with 46±10 cm) at cluster NW+ where it rose from 27 cm before to 63 cm after flooding (November 2009 and 2010, respectively). After flooding, the water levels ranged between 4 and 65 cm with a mean of 35±13 cm (Fig 3a).

Both TOC and TNb in the peat water increased significantly in the first year after flooding (linear regression, n = 102, TOC: R^2^ = 0.404, p<0.001, TNb: R^2^ = 0.368, p<0.001; Fig 3g and 3h). Concentrations of TOC rose by a factor of six, and TNb by a factor of four. Peat water concentration of TOC was 80±60 mg l^-1^ whereas concentration of TNb averaged at 5±3 mg l^-1^. Highest values of TOC and TNb occurred at cluster NW-, especially in late summer when overall concentrations were highest (Fig 3g and 3h).

Electric conductivity remained relatively constant whereas concentrations of Cl^-^ and SO_4_^2-^ dropped to three quarters and one third of pre-flooding conditions, respectively. Electric conductivity in the peat water was 6.0±2.7 mS cm^-1^ and concentrations of Cl^-^ ranged from 0.01 to 1.10 g l^-1^, averaged at 0.88±0.14 g l^-1^, while the concentrations of SO_4_^2-^ ranged from 0.01 to 11.0 g l^-1^ and averaged at 0.37±0.27 g l^-1^ (Fig 3d–3f). Across the whole range of values the relationship between electric conductivity and the concentrations of Cl^-^ and SO_4_^2^ was rather weak (linear regression, n = 118, R^2^ = 0.2663, p<0.001). However, up to values of 4 mS cm^-1^ electric conductivity was increasing together with the concentrations of Cl^-^ and SO_4_^2^ whilst above 4 mS cm^-1^ the concentrations of Cl^-^ remained constant or decreased and SO_4_^2-^ concentrations increased further whilst displaying strong statistical spread. Generally, the electric conductivities and the concentrations of SO_4_^2-^ in peat water were significantly higher in the SE of the study site compared to the NW while concentrations of Cl^-^ were homogeneous across the study site (see Table 1).

**Fig 4:**
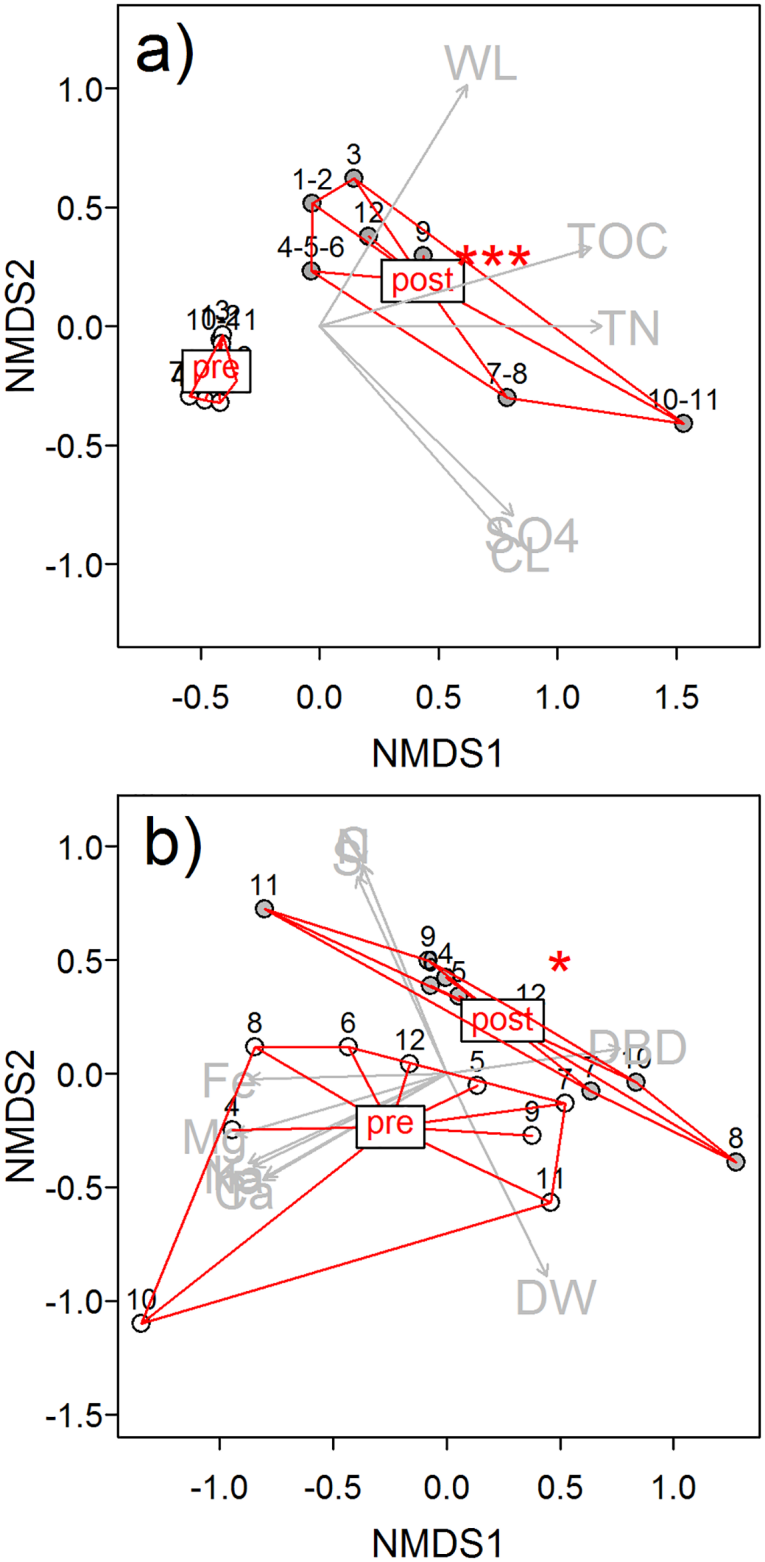
Properties of the a) peat water and b) peat (0-20cm) before (pre) and after (post) flooding. Spots which shared one dip well are represented by one data point. Only parameters that contributed significantly to the NMDS (p < 0.01) were included. Parameters included in peat water NMDS were water level (WL), the contents of total organic carbon (TOC) and nitrogen(TN), chloride (CL) and sulfate (SO4). Stress of the peat water NMDS was 0.012. Flooding significantly (p<0.001***) altered peat water characteristics. For pre-flooding peat water properties from the 25.11.2009 and for post-flooding those from the 25.11.2010 were used (n = 14).

After flooding, peat water properties became more heterogeneous (Fig 4a) and the SE clusters experienced the strongest change in peat water composition (Fig 3d–3h). At SE+ the increase in TOC and TNb was three times higher compared to all other spots and electric conductivity, Cl^-^ and SO_4_^2-^ even increased compared to pre-flooding conditions. Actually, highest concentrations of SO_4_^2-^ were found in dip wells P4 (SE-; median concentration of 0.78 g l^-1^ SO_4_^2-^) and P7 (SE+, median concentration of 0.71 g l^-1^ SO_4_^2-^), and extremely high values of both Cl^-^ (4–6 g l^-1^) and SO_4_^2-^ (4–10 g l^-1^) occurred from September to December 2010 (Fig 3e and 3f).

Parameters included in peat NMDS were dry bulk density (DBD), dry weight (DW), and contents of total carbon (C), nitrogen (N), sulfur (S), phosphorus (P), potassium (K), magnesium (Mg), calcium (Ca), sodium (Na), and iron (Fe). Stress of the peat NMDS was 0.041. Flooding altered peat water characteristics significantly (p<0.05*). Spots 1–3 were excluded from the peat property analysis due to missing values before flooding. For pre-flooding peat properties from the 25.11.2009 and for post-flooding those from the 23.08.2010 were used (n = 18).

#### Peat properties

The peat from the uppermost 20 cm had—on average—a dry bulk density (DBD) of 0.8±0.2 g cm^-3^, a content of organic matter (OM) in the dry mass of 68±11% and a water content of 77±5%. The C:N ratio averaged at 15.7. We observed the formation of FeS (black film on study site equipment) and H_2_S (smell) during field sampling campaigns. The DBD was relatively homogeneous throughout the study site. The contents of organic matter, the C:N ratio as well as the concentrations of C, N, S, P, K, Mg, Ca, and Na were higher in the NW compared to the SE of the study site (one tailed paired Wilcoxon tests, n = 96, p<0.05).

There was a trend to higher amounts of C, N, and S in and higher dry bulk density of the peat after flooding compared to pre-flooding conditions (Table 2). However, the twelve measurement spots varied with regard to their peat properties before and after flooding (Fig 4b). As single parameter flooding significantly increased concentrations of C, N, S and Fe (one-tailed paired Wilcoxon rank sum test, n = 84; p(C) < 0.05, p(N) < 0.05, p(S) < 0.01, p(Fe) < 0.05). Most remarkable, however, was the increase in dry bulk density after flooding (DBD (pre-flooding) = 0.6±0.2, DBD (post-flooding) = 0.8±0.1 g cm-3; one-tailed paired Wilcoxon rank sum test, n = 108, p < 0.001). In the peat zone with roots (D1, 0-10cm depth) dry bulk density doubled and in the more humified peat (D2, 10-20cm depth) it increased by factor 1.25. Additionally, the water content in D1 increased at half of the spots and decreased at the other half. Thus, on average water content did not change when comparing pre- to post-flooding conditions. In the lower peat layer (D2), however, water content increased after flooding, except at the spots 7 and 8 which were located on a sand lens.

**Table 2:** Peat properties before (“PRE”) and after (“POST”) flooding in the peat zones dominated by living roots (0–10 cm, D1) and the more humified peat (10-20cm, D2).

	n	PRE						POST				
		0-10cm			10-20cm			0-10cm			10-20cm	
**DBD**	84	0.38	(0.1)	<***	0.69	(0.1)	<***	0.78	(0.2)	ns	0.84	(0.1)
**OM**	47	71.36	(3.8)	>*	53.10	(19.3)	**ns**	64.88	(5.9)	ns	54.90	(14.8)
**C:N**	84	15.78	(0.6)	<*	17.10	(1.3)	**ns**	16.37	(1.1)	<**	18.24	(1.4)
**C**	84	28.04	(6.8)	>*	23.86	(9.0)	<*	35.02	(4.4)	ns	33.90	(6.4)
**N**	84	1.76	(0.3)	>*	1.41	(0.5)	<*	2.13	(0.3)	>**	1.9	(0.4)
**S**	84	1.11	(0.3)	>**	0.92	(0.3)	<**	1.42	(0.3)	ns	1.51	(0.8)
**P**	84	3.75	(1.6)	>***	1.40	(0.7)	**ns**	2.55	(0.4)	>**	1.95	(1.1)
**K**	84	3.85	(1.9)	>**	2.00	(0.5)	**ns**	4.30	(2.0)	>**	3.15	(1.7)
**Ca**	84	14.70	(3.5)	>*	12.05	(4.5)	**ns**	13.90	(1.6)	ns	14.75	(2.7)
**Na**	84	25.90	(16.3)	>***	12.75	(6.4)	**ns**	19.78	(10.6)	ns	26.10	(20.1)
**Mg**	84	12.25	(6.3)	>*	8.30	(4.3)	**ns**	12.35	(5.8)	ns	15.30	(10.5)
**Fe**	84	23.90	(4.6)	**ns**	20.60	(5.7)	**ns**	25.90	(4.4)	ns	28.81	(9.7)

Medians and median deviations (median (md)) are presented. Direction of significance testing and level of significance are indicated by: ns (p>0.05),

* (p<0.05),

** (p<0.01) and,

*** (p<0.001).

OM = percent organic matter in %, DBD = dry bulk density in g cm-3, C:N = C:N-ratio, C = total carbon in %, N = total nitrogen in %, S = total sulfur in %, P = total phosphorus in g kg-1 dry weight, K = total potassium in g kg-1 dry weight, Mg = total magnesium in g kg-1 dry weight, Na = total sodium in g kg-1 dry weight, Ca = total calcium in g kg-1 dry weight, Fe = total iron in g kg-1 dry weight.

Neither flooding nor dominant vegetation nor peat layer had a consistent effect on all peat properties (Fig 4b, see Table 2 for an overview of peat properties before and after flooding). Regarding the entity of all recorded peat properties (permutation test of NMDS fit, 1000 permutations, p<0.05), the peat zone with roots (D1) was significantly different from the peat layer below that zone (D2).

### Methane fluxes

**Fig 5:**
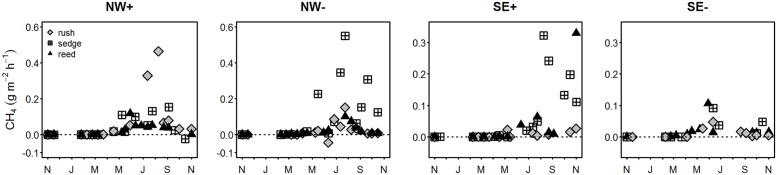
Exchange of CH_4_ at the more (NW+, SE+) and less (NW-, SE-) inundated clusters. The CH_4_ exchange was measured from November 2009 until November2010 using the closed chamber approach.

From November 2009 to November 2010, in total 267 fluxes of CH_4_ were recorded; of which 39% were excluded from further analysis because they did not meet the quality criteria. On average 260±60 g m^-2^ CH_4_ were emitted from the study site during the measurement period. The largest share was released during summer and autumn (98±3% of total annual). From June to November 2010, peak emissions between 226.4 and 727.5 mg m^-2^ h^-1^ occurred, but in general CH_4_ fluxes ranged from -43.8 to 727.5 mg m^-2^ h^-1^. During the first year after rewetting, reed, rush, and sedge stands emitted, 200±50 g m^-2^ CH_4_, 300±140 kg m^-2^ CH_4_, and 470±140 kg m^-2^ CH_4_, respectively. CH_4_ fluxes increased significantly (linear regression, n = 162, R^2^ = 0.1152, p < 0.001) after flooding. In November 2010, fluxes of CH_4_ were on average 186-times higher than in November 2009 (one-tailed Wilcoxon rank test, n = 48, p < 0.001). Furthermore, CH_4_ fluxes from the NW clusters were higher compared to the SE clusters (one-tailed Wilcoxon rank test, n = 162, p < 0.05) (Fig 5) during the period of study.

### Methane exchange in relation to controls

#### Controls of single methane fluxes during the first year after flooding

The GAM- interpolations for the environmental variables had a mean percentage standard error of 0.5%. Due to only one data point in autumn 2009 and small fluxes in winter 2009 (-1±9 μg m^-2^ h^-1^), mean percentage standard error was 266% for CH_4_. For the rest of the study period mean percentage standard error for CH_4_ was 3%.

**Fig 6:**
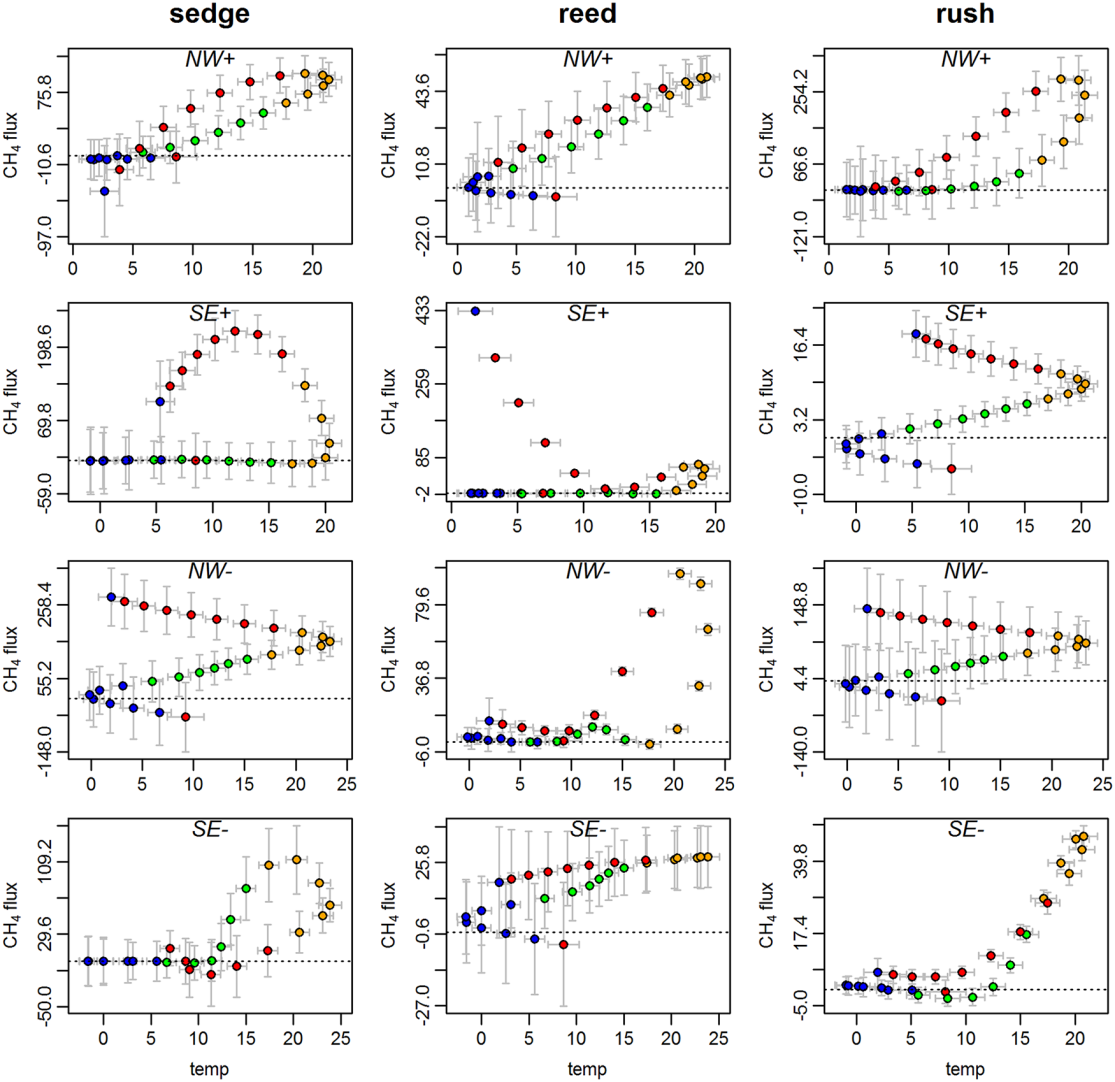
Impact of peat temperatures (°C) on CH_4_ fluxes (mg m^-2^ h^-1^) in the four seasons (blue = winter, green = spring, yellow = summer, red = autumn) in the northwest (NW) and the southeast (SE) of the study site at the more (+) and less (-) inundated measurement spots. Data are interpolated by generalized additive models. Error bars indicate the standard error of the interpolation.

Fluxes of CH_4_ significantly increased during the first year after flooding (linear regression, n = 28, p < 0.001, R^2^adj (CH4) = 0.2496). Interpolated CH_4_ fluxes were 205-times higher in the autumn after flooding compared to pre-flooding conditions (one-tailed Wilcoxon rank test, n = 33, p < 0.001). The increase in CH_4_ fluxes was positively related with the increase in concentrations of TOC and TNb in the peat water (linear regression, p<0.001, n = 336, R^2^adj(TOC) = 0.2073; R^2^adj(TNb) = 0.1436). The highest CH_4_ fluxes recorded in this study occurred in late summer on sedge spots (Fig 5) and (interpolated) CH_4_ fluxes from sedge spots were significantly higher compared to those from rush and reed spots (pairwise Wilcoxon rank tests, n = 28, p< 0.01). The season—and, thus, the development of vegetation and the progression of flooding—influenced the relationship between the controlling parameters and CH_4_ exchange. For example, at the same peat temperatures the fluxes of CH_4_ were higher in autumn compared to spring 2010 (Fig 6). Although the patterns at individual spots differed from or even contradicted the general trend, some similar trends occurred within clusters (e.g., NW+ in Fig 6) or for one plant species (e.g., rush in SE+ and NW-).

#### Controls of total atmospheric methane exchange in the first year after flooding

**Fig 7:**
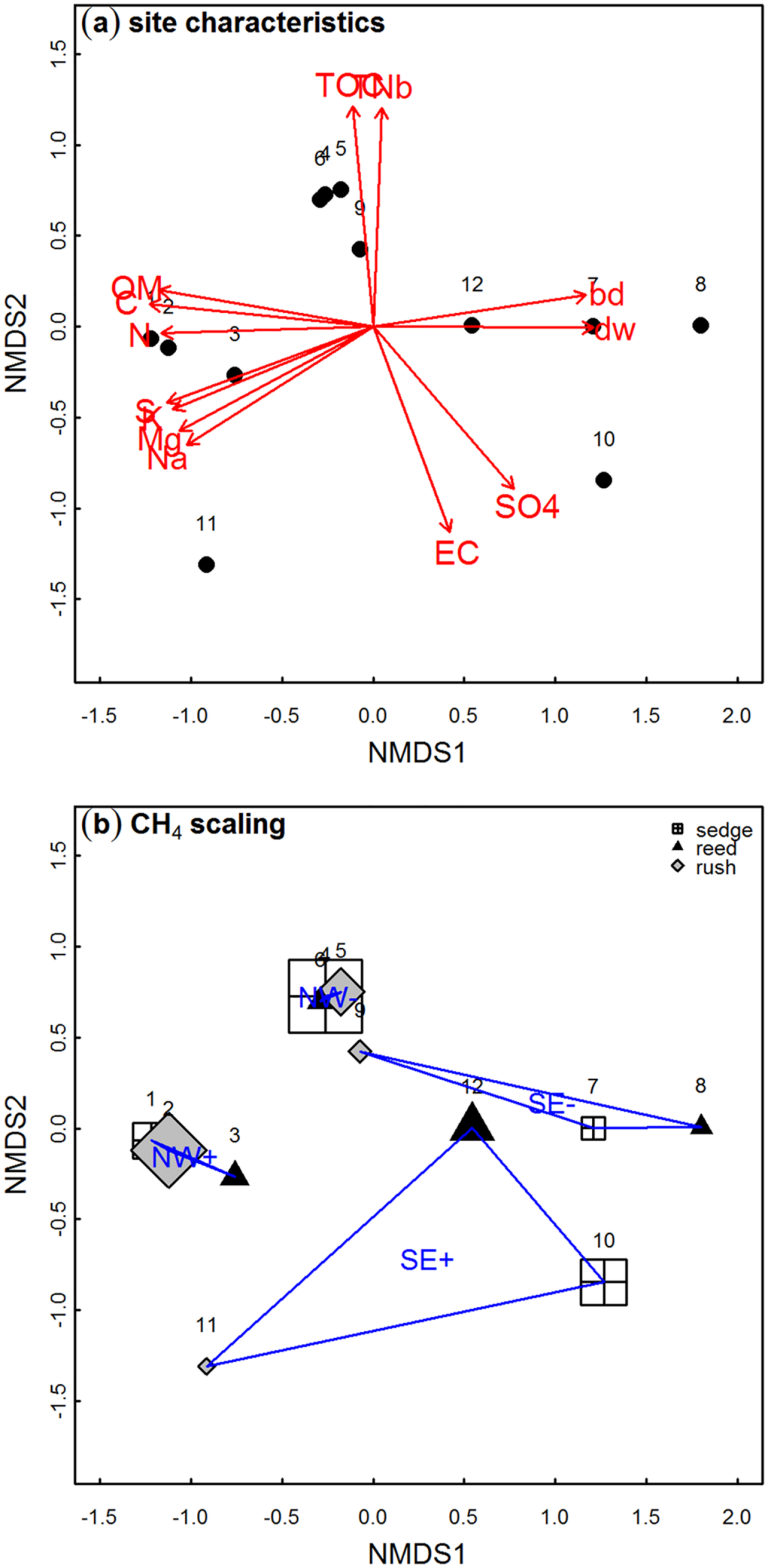
Interaction of site characteristics with the exchange of CH_4_ in the first year after flooding. Measurement spots are arranged according to Bray-Curtis-dissimilarity regarding the average site characteristics (median) of the measurement spot (for abbreviations see Fig 4) (a). The size of the symbols represents the relative values of the annual emissions of CH_4_ (b). All parameters contributed significantly (p<0.001) to the ordination. The polygons display the cluster (i.e. location and inundation level, see Fig 1c) of the particular measurement spot. Clusters differed significantly regarding their site characteristics (p<0.05). Stress of the NMDS was 0.012.

According to the final NMDS ordination TOC, TNb, dry bulk density, dry mass content (100-water content), electric conductivity, peat water sulfate content, concentrations of S, K, Mg, Na, C, and N as well as content of organic matter contributed significantly to the characterization of the environmental conditions at the 12 measurement spots (permutation test of NMDS fit, 1000 permutations, p<0.001; Fig 7a). The environmental variables and peat properties of the four clusters differed significantly from each other (permutation test of NMDS fit, p<0.001) although the measurement spots within clusters NW+ and NW- were quite homogeneous. On the contrary, the site characteristics of the spots within clusters SE- and especially SE+ differed strongly from each other (Fig 7b and 7c).

We could not find any relation to the amount of atmospheric exchange of CH_4_ when testing the site characterizing parameters from the ordination plus dominant vegetation by a permutation test of the ordination. Nevertheless, we found high annual emissions of CH_4_ from single spots irrespective of water and peat properties, of the cluster and of dominant vegetation; but there seemed to be a trend towards higher emissions of CH_4_ from sedge (Fig 7b). According to linear regression (n = 12, p < 0.05), annual emissions of CH_4_ were higher when the cover of rooted vegetation was lower, but this was not significant (R^2^ = 0.189).

## Discussion

### Water level increase and die-back of vegetation

Rewetting of the coastal peatland was achieved by blocking the outflow of the catchment with a ground sill. This turned the previously summer dry fen into a shallow lake with a mean water level of 35±13cm and thereby triggered a change in the characteristics of the ecosystem. Strength and direction of the change differed considerably, depending on the starting conditions of the single measurement spots.

Most obvious was the die-back of rooted vegetation that was especially substantial in sedge stands in which as few as 0–20% of the measurement spots were covered by sedge post-flooding (compared to over 50% pre-flooding). A strong decrease in sedge cover under high inundation is also reported by Timmermann et al. [51] from a percolation fen in Western Pomerania. Such strong die-backs after flooding might be predictable when the plant physiology of the respective species is well known, e.g., [52, 53]. Short grass species are known to suffer most from the short-term negative effects of flooding [54] and are less able to recover [55]. According to Kvet (1984; in [56]), the growth of wetland sedges (*Carex* spp.) is favored by low inundation levels and hampered by high inundation. On the other hand, large emergent macrophytes like *Phragmites australis*, that are highly tolerant to flooding [57], showed only small decrease after flooding. Thus, it is to be expected, that adapted wetland plants will gain dominance under the new conditions [35]. There are both reports about strong fluctuations and higher variability in the first years after rewetting [58] as well as about homogenization in vegetation cover under flooding [59]. Nevertheless, there seems to be an element of chaos in these succession trajectories: Reviewing the literature on restoration of wetlands, Klötzli & Grootjans ([58], p.210) state, that “although many species fluctuations could be explained after identification of changes in environmental conditions […], many other fluctuations were quite unforeseeable […]”.

### Water level increase and changed peat and peat water properties

The variation in peat water and peat properties across the study site became stronger after flooding. The only consistent effect of flooding was the approximate doubling of dry bulk density that was most likely due to the (tripled) weight of the water column, which likely led to compression of the peat. This effect was especially pronounced in the uppermost 10cm dominated by living roots.

Despite the variations between the measurement spots, there was an overall trend to increased concentrations of C, N, and S in the peat as well as of TOC and TNb in the peat water. The latter we observed especially at low inundation levels and it was most pronounced where vegetation suffered from the heaviest die-back (cluster NW-). Schulz et al. [56] found that helophyte species like e.g. *Carex riparia* or *Phragmites australis* store considerable amounts of carbon and temporarily withdraw high amounts of nutrients from the top soil during the growing season; elements that will be released when vegetation dies back. Zak et al. [28, 60] report that drained fens may lose their nutrient sink-function during the initial stage of rewetting. They have observed increased amounts of redox sensitive substances and enhanced availability of decomposable organic matter in the upper, highly decomposed peat horizon that caused mobilization of P, organic carbon and ammonium in the soil and surface water of rewetted fens, causing nutrient enrichment. Therefore, we attribute the increase of C and N in the water and peat to the decay of inundated plants although we are aware that we measured total concentrations only. The average C:N-value of 15.7 is characteristic for eutrophic conditions, that are related to the occurrence of easily degradable organic matter by Succow and Joosten [17]. Thus, this C:N-value could indicate ample supply with fresh and easily degradable C- and N-compounds (e.g., hemicelluloses [61]) that could fuel microbial activity. In accordance, Hahn-Schöfl et al. [62] observed the formation of a new sediment layer from easily decomposable plant litter which originated from reed canary grass killed by flooding a degraded fen.

In addition, flooding reduced the brackishness of the fen by diluting the concentrations of Cl^-^ and SO_4_^2-^ in the peat water. Still, the mean values of 0.88 g l^-1^ Cl^-^ and 0.37g l^-1^ SO_4_^2-^ were small compared to concentrations in Baltic Sea water (5–6 g l^-1^ Cl^-^ and ca. 0.6 g l^-1^ SO_4_^2-^ (own measurements in December 2010 and [63])) and to average ocean water (ca. 19g l^-1^ Cl^-^ and 3 g l^-1^ SO_4_^2-^ [64]).

### Water level increase and increased methane emissions

We recorded a strong increase in the emission of CH_4_ compared to pre-flooding-conditions. CH_4_ emissions—on average—increased 186-fold from pre- to post-flooding conditions. Pre-flooding mean CH_4_ efflux was 0.0014 ± 0.0006 kg CH_4_ m^-2^ a^-1^ in 2009 [34]. In the first year after flooding, average CH_4_ emission was 0.26±0.06 kg CH_4_ m^-2^ at comparable spots. Due to our set quality criteria for fluxes we omitted a relatively high share of fluxes from later analysis (39%). Many of these were likely ebullitive fluxes. Thus the very high annual CH_4_ exchanges we find can be seen as conservative estimates and the real CH_4_ emissions were likely even higher than reported here. Although there might as well be a contribution of inter-annual variation in climatic conditions and some effect of variation in location of measurement spots, this is quite a dramatic increase that led to an exceptional annual emission that compares well to the highest values in the literature that are reported from tropical floodplain wetlands in Costa Rica (up to 0.35 kg CH_4_ m^-2^ a^-1^; [65]). Our values (e.g., 0.47±0.14 kg CH_4_ m^-2^ a^-1^) for sedge spots are even higher than those and are by far higher than the highest estimates that Saarnio et al. [66] suggest for fresh marshes (0.091 kg CH_4_ m^-2^ a^-1^), saltwater marshes (0.076 kg CH_4_ m^-2^ a^-1^) or minerotrophic mires (0.048 kg CH_4_ m^-2^ a^-1^) based on a comprehensive review of the relevant literature from Europe.

Couwenberg et al. [32] compiled studies that report very high CH_4_ emissions and state that specific (starting) conditions such as emissions from ditches only [67], flooded harvest [68], or compacted gyttja soils [21] may explain such very high CH_4_ emissions. Similarly, Hahn-Schöfl et al. [62] found the formation of fresh (2.5years old) plant litter to be the source for large fluxes of CH_4_ and CO_2_ after flooding a degraded fen grassland. The presence of species with well developed aerenchyma may be another possible explanation for high CH_4_ emissions [32, 69]. At our study site, the presence of plants with aerenchyma tissue seems to have had minor influence because we measured high CH_4_ emissions especially from plots where total vegetation cover has decreased strongly after flooding. Thus, we infer that the supply with fresh substrate from plant species that could not outgrow the increased water levels (i.e., sedges) was the main source and, thus, the major driving force for the exceptionally high CH_4_ emissions after flooding at our study site. The concomitant increase of TOC and TNb in the peat water, the accumulation of organic matter, N, P, and K support this hypothesis.

The rewetting of the “Rodewiese” was conducted by flooding the fully vegetated site. Thus it was in sharp contrast to Couwenberg et al.’s [32] recommendation for minimizing GHG emissions after peatland rewetting, i.e. the peatland should be free of lush and abundant vegetation and the water table should be raised to a level constantly close to the surface (±10cm). According to their GEST classification an annual GHG release of 1t CO_2_-eq. ha^-1^ a^-1^ was expected from the study site (water level +6, eutrophic, sub-neutral). Taking into account that Koebsch et al. [39] reported a sink for CO_2_ of approx. 11 t ha^-1^ for the vegetation period, we could also assume conservatively that the site is neutral with regard to the exchange of CO_2_ with the atmosphere when considering the whole year. Then still, the average CH_4_ exchange alone represents a GWP of approx. 73 t CO_2_-eq. ha^-1^ a^-1^ (calculated with CH_4_ having a radiative forcing of 28*CO_2_ [70]). This confirms Couwenberg et al.’s [32] remark that the GESTs are only reliable for the mid-term-perspective after 3 to 5 years. Nevertheless, we think, that these high initial emissions should be considered in GHG balances of rewetting projects.

### Complex modulation of increased methane emissions after flooding

#### Methane and temperature

On our study site neither the variation of the annual estimates nor of the single fluxes of CH_4_ could be attributed to a consistent set of control parameters. In accordance with DeLaune et al. [71] CH_4_ emissions from all spots were highly variable, both within a cluster and over time, but they were at least partially correlated with temperature. Given anoxic conditions, temperature is one of the most important controls on CH_4_ exchange in wetlands [72, 73, 33]. Kayranli et al. [24] even designate temperature the most important control factor outside the growing season during which organic matter availability primarily limits methanogenesis [72, 73, 33]. Similar to Bartlett et al. [74] we found that although CH_4_ fluxes were related to peat temperature (based on fortnightly, interpolated values), other variables created higher fluxes in autumn than in spring for equivalent temperatures (Fig 6). Studies from salt marshes have reported similar temporal trajectories regarding rates of bacterial sulfate reduction [75] and CO_2_ production [76]. The availability of readily metabolized organic substrates as marsh plants mature and die was hypothesized to cause the higher rates in autumn. In addition, the authors infer that the modulation of the processes must be located in the root zone. This explanation suits to our results and is supported by the findings of Tuittila et al. [77] on the seasonal dynamics of CH_4_ exchange in the first three years in a rewetted cut-away peatland. In addition to soil temperature (at 15 and 30cm depth) and an effective temperature sum index they included cotton-grass cover (*Eriophorum vaginatum*) as well as the interaction between cotton-grass cover and water level in their model. This model explained 81% of the variation in the CH_4_ flux data. Overall, they also arrived to the conclusion that increased primary production and the consequent deposition of substrate to anoxic conditions caused higher CH_4_ emissions in the first three years after rewetting.

#### Methane and seasonality

Tuittila et al. [77] hypothesize that at the end of the vegetation period plants respire less of the fixed carbon and, thus, root exudates and plant residues produced during high season are accumulated as substrate for later methanogenesis. Thus, although instantly available labile organic matter seems to stir up significant inorganic production of CO_2_ and CH_4_ in (moist) peat (e.g., [62]), substrate supply is highest when the vegetation period is beyond its zenith [78]. This is well in accordance with our findings, that (i) the annual estimates of CH_4_ exchange were largely fueled by the peak emissions of CH_4_ that were recorded in late summer; and (ii) the highest CH_4_-fluxes that were recorded during the study (727 mg m^-2^ h^-1^) occurred in late summer 2010 at the sedge stands of cluster NW-, where vegetation suffered from the heaviest die-back and the overall values of TOC and TNb were highest. This highest CH_4_-flux of 727 mg m^-2^ h^-1^ is 7-times higher than the highest rates reported by Heyer and Berger [79] from a shallow coastal area of the Baltic Sea, between the islands of Rügen and Hiddensee. The average water depth of 25-35cm in that study was in the range of our study site but the organic matter content of the sediment in 0–10 cm depth (2.6–8.8% of dry weight) was much lower than the 68% in our study site. The authors note, however, that organic matter content increased considerably after an irregular seasonal transport of organic matter (benthic algae, seaweeds) into the study area and the formation of microbial mats but unfortunately they did not quantify organic matter afterwards (ibid.). Nevertheless, Heyer and Berger [79] found the amount of organic matter in the sediment to be the crucial factor for the inter-annual and seasonal variations of CH_4_ emissions and also for small-scale spatial differences.

#### Methane and trophic state of the wetland ecosystem

We have already discussed the strongly increased input of fresh litter after flooding and concluded that freshly synthesized labile organic matter due to vegetation die-back may well have been driving the high CH_4_ emission from our study site. However, we cannot attribute variations in CH_4_ emissions to it statistically. Admittedly this might be due to our low number of replicates in a peatland with a high inherent variability of site characteristics. It seems noteworthy, though, that we have measured the highest CH_4_ fluxes in the NW of our study site that was characterized by higher concentrations of organic matter, nutrient elements like C, N, and P, and concentrations of TOC and TNb. Keeping in mind that “the relationship between increased nutrients and flux does not appear to be simple” [74] we might interpret this as an indication for CH_4_-modulation via concentration of organic matter and nutrient elements. This would be well in agreement with the suggestion that the trophic status of the water and the sediment may be an important factor regulating emissions of drainage ditches and lakes [67]. By using multiple linear regression the authors were able to explain 87% of the variation in CH_4_ fluxes by PO_4_^3-^ concentration in the sediment and Fe^2-^ concentration in the water, and 89% of the CO_2_ flux by depth, EC and pH of the water.

In our study site, the SE clusters were more dominated by electric conductivity and higher concentrations of Cl^-^ and SO_4_^2-^. Here the CH_4_ fluxes were lower. Therefore, another explanation of the high variability in CH_4_ efflux might be the sulfate concentration. According to Poffenbarger et al. [80] our study site is at the threshold below which variation in porewater CH_4_ increases dramatically with an average of 0.4 g l^-1^ SO_4_^2-^. Taking into account that CH_4_ in the pore water is likely the source for CH_4_ emission [81], variation in the former may explain variation in the latter. Furthermore, Poffenbarger et al. [80] report that sulfate depleted clusters might form where input of labile organic carbon is high. This, in turn, might facilitate the production and consequently the release of CH_4_ by reducing substrate competition and establishing favorable redox-conditions [82]. During field campaigns, we regularly observed FeS-films on our study site equipment and the smell of H_2_S both of which indicates the presence of sulfate reduction. Furthermore, a linear regression of CH_4_ emissions against SO_4_^2-^concentrations in the peat water shows a relatively close relationship (R^2^ = 0.4538, p<0.001). Therefore, we hypothesize the co-existence (e.g., [83, 80]) and simultaneous stimulation of sulfate reduction and methanogenesis by excess substrate supply through decaying vegetation.

#### Methane and plant species

Poffenbarger et al. [80] point out that plants are a major source of variation in CH_4_ emissions because they are sources of both organic carbon and certain electron donors. Before flooding, Koebsch et al. [34] found vegetation to be the major controlling factor of CH_4_ emissions from our study site. Similarly, Tuittila et al. [77] report that the CH_4_ dynamics in a restored cut-away peatland were mainly controlled by vegetation succession of the typical dominant species (cotton-grass). Only recently Bhullar et al. [84] have confirmed that plant species composition influences CH_4_ emission from wetlands, and suggest that they should be considered when developing measures to mitigate GHG emissions. This seems all the more important when taking into consideration that plants can be a control for CH_4_ production even when they are no longer growing on the site: In their study of peatland ecosystems in North America Yavitt et al. [85] have found the highest CH_4_ production at a bog site where sedges had been growing in the recent past and the decomposition of sedge residues in the peat below the surface supported CH_4_ production. We find this partly confirmed in our study as we saw a trend to highest CH_4_ emissions from spots that were dominated by sedges before and by floating vegetation after flooding.

#### Methane and site heterogeneity under flooded conditions

Heterogeneity of the site characteristics was medium before and large after flooding as common in ecosystem research and particularly in rewetting studies [10]. For instance, in their review on CH_4_ release from European wetlands and watercourses Saarnio et al. [66] state that uncertainties of the CH_4_ release estimates did not only arise from uncertainties in the estimation of the area of ecosystem types but also from their internal heterogeneity. For our study site, Koch et al. [35] have confirmed the influence of spatial heterogeneity on annual estimates CH_4_ emissions from reed stands. Furthermore, Saarnio et al. [66] note that their reviewed publications did not support a more detailed analysis of the dependence of CH_4_ release on different abiotic and biotic factors.

In our study, the selected clusters differed significantly in their peat and water properties—apart from the highly individual behaviour of the twelve measurement spots. The spots 7 and 8 (SE-, sedge & reed, dip well P4) were special cases: Here, the peat and water properties differed considerably from the other spots (increased TOC, TNb, EC, Cl^-^ and SO_4_^2-^, decreased nutrient concentrations after flooding). This was most likely due to the location of these spots on sand lenses which are remainders from land amelioration. Maybe a larger sample size might have diminished the influence of these spots on the data set and led to a clearer picture of the ongoing changes; but under the prevailing conditions the installation of more measurement spots was impossible and we wanted our setup to reflect the real heterogeneity of the study site.

In addition to heterogeneous site characteristics, the process of flooding itself is highly dynamic and affects an ecosystem at different levels. In our study, the interaction between season and flooding could have masked relationships between environmental variables and atmospheric CH_4_ exchange. Furthermore, there were interactions of site characteristics themselves. For instance, we found different responses of particular plant species to flooding that possibly influenced peat properties which directly or indirectly may have affected CH_4_ exchange. Furthermore, high temperatures in late summer could have enhanced decomposition of plant species that could not outgrow the increased water level and thereby maximize CH_4_ emissions.

Therefore, it was difficult to assign changes of peat and water properties or CH_4_ exchange to the factor flooding only. Moreover, it is not surprising that it was not possible to disentangle the interactions of peat and water properties with the exchange of CH_4_ in the first year after flooding. Kaat & Joosten [2] stated that the effect of increased CH_4_ emissions after rewetting is usually of short duration, and rewetting of peatlands always leads to a net reduction of climate relevant emissions on the mid and long-term. Therefore, it seems not reasonable to evaluate the overall success of a peatland restoration regarding atmospheric CH_4_ exchange and ecosystem properties based on data gathered directly after rewetting alone. Instead, it is essential to monitor the further development to properly evaluate the effect of flooding of the examined fen; especially regarding the amount, species and direction of GHGs which are exchanged and the shift of the ecosystem parameters such as vegetation cover.

## Conclusions

In the short term perspective covered in this study, i.e. first year after flooding, our hypotheses were confirmed: Rewetting by flooding was not beneficial in order to reduce GHG emissions, especially due to the very strong increase in CH_4_ emissions. This effect was even enhanced by the fact that the study site was a negligible source of CH_4_ and likely neutral with respect to CO_2_ exchange before rewetting by flooding. In the present case the installation of the ground sill targeted not only at the restoration of the biogeochemical functioning of the ecosystem but the focus was on establishing suitable habitat for waterfowl and other faunal elements that need shallow open water. This suggests that it is necessary to carefully evaluate the advantages and disadvantages and the possible implications of rewetting projects for different ecosystem properties.

Furthermore, we observed an overall destabilization of the ecosystem functioning: The environmental parameters that are commonly used to explain variation in GHG exchange did not show any consistent correlation and some showed dramatic changes when comparing pre- and post-flooding. This kind of chaotic reaction of many ecosystem properties makes general conclusions about the effect of flooding difficult. However, our study is the first that demonstrates in detail the de-stabilization of a peatland ecosystem after rewetting. Therefore, we think, it gives valuable insights into the ecosystem functioning of rewetted peatlands despite its limitations.

Our results suggest that rewetting projects should be monitored not only with regard to vegetation development but also with respect to biogeochemical conditions. Further, high CH_4_ emissions that likely occur directly after rewetting by flooding should be considered when forecasting the overall effect of rewetting on GHG exchange of a particular site. After all, it seems reasonable to state, that it is not expedient to evaluate the success of peatland restoration regarding atmospheric C-exchange and ecosystem properties based on data gathered shortly after rewetting only. Instead, our results illustrate the necessity for more research on the effect of rewetting on intensively drained, degraded temperate fens. Especially, longer term comprehensive monitoring data of the development of biogeochemical processes and vegetation patterns after rewetting are needed to foster our understanding of the functioning of restored peatlands to better advise the planning of rewetting measures.

## Supporting Information

S1 Dataset Concentrations of CH_4_ within the measurement chambers at the biweekly sampling campaigns during the first year of flooding. (CSV)

S2 Dataset Peatwater properties during the first year of flooding. (CSV)

S3 Dataset Peat properties before and after flooding. (CSV)
